# Supplementary material for: Acoustic and language-specific sources for phonemic abstraction from speech
Source: Nat Commun. 2024 Jan 23;15:677. doi: 10.1038/s41467-024-44844-9 (PMC10805762; doi:10.1038/s41467-024-44844-9)
Supplement: Supplementary file 1 — Supplementary Information [file 41467_2024_44844_MOESM1_ESM.pdf]

Acoustic and language-specific sources for phonemic abstraction from speech

Anna Mai,[1*] Stephanie Riès,[2,3] Sharona Ben-Haim,[4] Jerry J. Shih,[5] Timothy Gentner[6,7,8]

[1]University of California, San Diego, Linguistics;
[2]San Diego State University, School of Speech, Language, and Hearing Sciences;
[3]San Diego State University, Center for Clinical and Cognitive Sciences;
[4]University of California, San Diego, Neurological Surgery;
[5]University of California, San Diego, Neurosciences;
[6]University of California, San Diego, Psychology;
[7]University of California, San Diego, Neurobiology;
[8]University of California, San Diego, Kavli Institute for Brain and Mind

Author Contact Information:

- Anna Mai, *acmai@ucsd.edu* (corresponding)

Supplementary Information

Supplementary Note 1

The central logic of the study requires coronal taps to be acoustically distinct from coronal stops, and requires there to be no significant acoustic difference between coronal taps based on their phonemic identity. This section demonstrates that this is indeed the case for the coronal stops and taps present in the Buckeye stimuli.

The primary acoustic feature that distinguishes coronal stops from coronal taps is their duration.[1–3] While voiced and voiceless American English stops are respectively roughly 70-90ms and 98-130ms in duration,[4–6] coronal taps last a mere 10-40ms.[1] Previous investigation into the neutralization of /t/ and /d/ in intervocalic environments has found that the variability in coronal tap duration is primarily dependent on the quality of the preceding vowel and not on the phonemic identity of the tap itself.[1] In fact, numerous studies have found that /d/ and /t/ taps do not differ significantly in their closure duration.[1,7–9] This finding is replicated in the stimuli used in this study, as shown in Supplementary Figure 1a. A one-way ANOVA indicated that there was a significant effect of phonemic category on phone duration [F(3; 1,666)=107.75, $p \leq 0.001$], and *post hoc* comparisons using the Tukey HSD test indicated that voiceless coronal stops in the stimuli are significantly longer than voiced stops, which

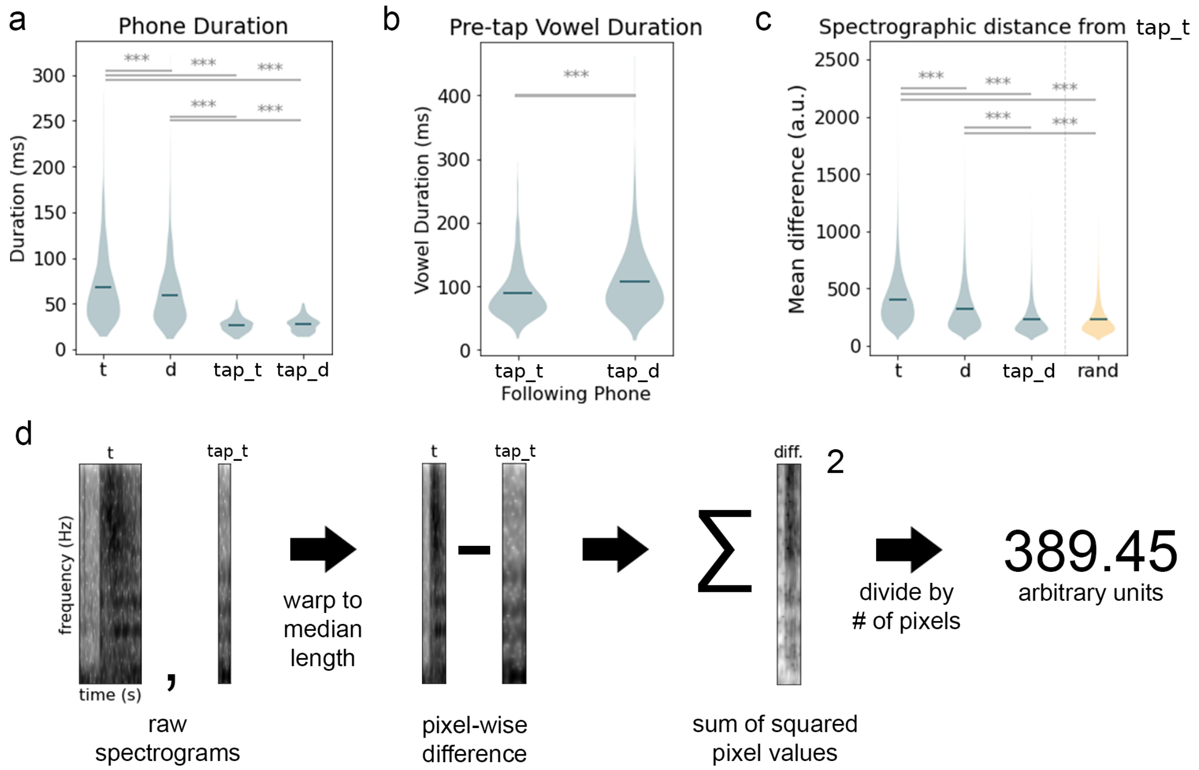

Supplementary Figure 1. **Coronal tap acoustics.** **a** Durations of coronal stops and taps in the Buckeye Corpus (n=1669). **b** Durations of vowels immediately preceding coronal taps in the Buckeye Corpus (n=1310). **c** Spectrographic distance of **t**, **d**, and **tap_d** sounds from **tap_t** (green); Spectrographic distance of a random split of all taps from the remainder of taps (yellow)(n=339845). **d** Schematic representation for the calculation of spectrographic distance. Distribution means are shown by the thick dark green lines in plots **a-c**, and significance levels are indicated on the following scale: * $p \leq 0.05$, ** $p \leq 0.01$, *** $p \leq 0.001$.

are in turn significantly longer than coronal taps, each at the $p \leq 0.001$ level. However, /d/ and /t/ taps do not differ significantly from one another in duration ($p = 0.9$). Thus, on the basis of their duration, coronal taps are distinct from coronal stops yet not distinct from one another on the basis of their phonemic identity, satisfying the study's foundational assumptions.

Nevertheless, the stimuli used in this study were taken from natural speech and undoubtedly vary along more acoustic dimensions than duration. As a means of further ensuring that the acoustic differences between coronal stops is greater than the difference between coronal taps, a measure of spectrographic distance was calculated for coronal stops and taps. First, wide-band spectrograms were created for all stimuli using Praat's default settings, and the spectrographic segments corresponding to coronal stops and taps were excised using the segmentation and labelling given in the Buckeye Corpus. The median duration of these segments was calculated (in bins) and all spectrographic segments were resized to this duration using the resize function from the transform module of the Python library `scikit-image`.[10] This function performs bi-linear interpolation to resize the image. Prior to down-scaling any images, it applies a Gaussian filter with a kernel size of $(s-1)/2$, where s is the down-scaling factor, to prevent any anti-aliasing artifacts. For each pair of resized spectrograms, the sum of the squared pixel-wise difference was calculated, and divided by the size of the spectrogram in pixels. This process is shown schematically in Supplementary Figure 1d. Supplementary Figure 1c plots the distances of [t], [d], and /d/-tap spectrograms from /t/-tap spectrograms (green), as well as the distances for a random split of all coronal taps (yellow). A one-way ANOVA indicated that there was a significant effect of phonemic category on spectrographic distance from /t/-tap [F(3; 339,842)=9124.47, $p \leq 0.001$], and *post hoc* comparisons using the Tukey HSD test indicated that the distance between /t/-tap and /d/-tap is significantly less than the distance between /t/-tap and either of the coronal stops at the $p \leq 0.001$ level. However, when mean distance is calculated for a random split of taps not based on phonemic identity, it is not significantly different from the mean distance calculated based on the phonemic identity of the taps ($p = 0.44$). Once again, this shows that the phonemic identity of coronal taps cannot be determined from the acoustic properties of taps themselves.

Although taps derived from /d/ and /t/ are acoustically indistinguishable from one another, the length of the vowel that precedes a tap systematically varies in duration based on the phonemic identity of the tap. When preceding a tap derived from /d/, vowels are approximately 10% longer than those preceding a tap derived from /t/.[1,2,9,11] This pattern is also observed in the Buckeye Corpus, where vowels preceding taps derived from /d/ were on average 18.31ms (SD=[13.23, 23.38]) longer than vowels preceding taps-derived from /t/ [t(1309)=50.16, $p \leq 0.001$] (Supplementary Figure 1b). Thus, there does exist an acoustic cue to the phonemic identity of taps. On this basis, one could argue that any observed difference in the neural response to medial /d/ and /t/ is due to the duration of the preceding vowel, seemingly undermining the foundational assumption of the study. For this reason, this study focuses on the 500ms following the onset of coronal stops and taps. By timelocking the analyzed response to the beginning of closure and using the preceding 100ms to baseline the signal, the acoustic impact of the preceding vowel is effectively neutralized. Though it remains possible that the duration of the preceding vowel cues the listener to the phonemic identity of the following tap, differences observed in the response /d/ and /t/ taps must be based on their categorization, and not the acoustics of the preceding vowel itself, because the mean signal in the 100ms preceding the tap is subtracted from the analyzed response, and /d/ and /t/ taps themselves do not differ acoustically from one another. In other words, though vowel duration may cue the phonemic identity of the tap, by timelocking the response to the beginning of the tap, any difference in response cannot be reducible to the preceding *acoustic* context.

Supplementary Note 2

Speech responsive electrodes were defined independently for each band, and across the ten subjects,

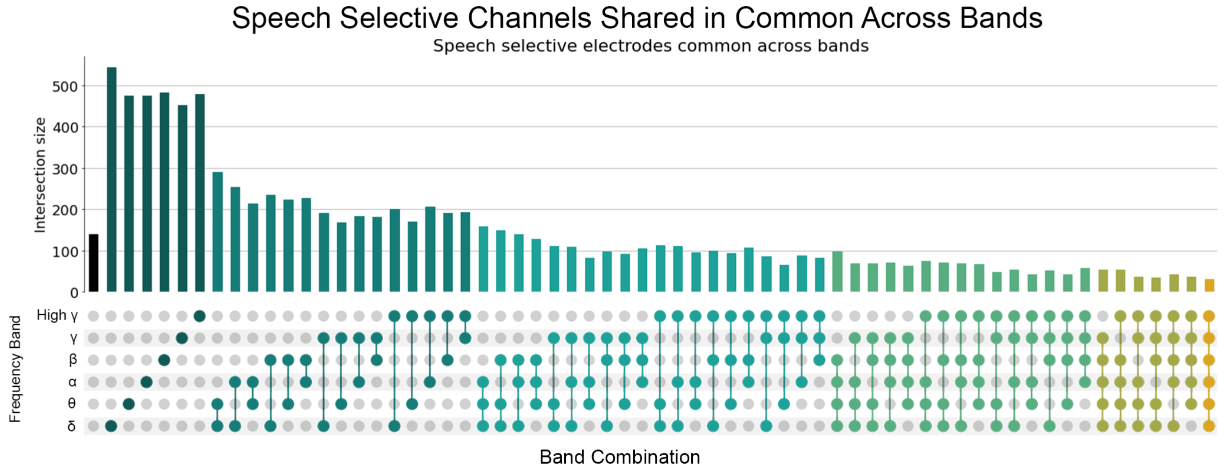

Supplementary Figure 2. **Degree of overlap across bands for channels defined as speech responsive.** Lower dot matrix plot indicates the combination of bands being considered, and the upper bar plot shows the number of speech responsive channels shared in common for that combination of bands. The left most column (black bar) shows the number of electrodes that were not responsive to speech for any band, and color indicates the number of bands in each combination.

1,215 (SE±28.1) electrodes were found to be speech responsive for at least one band, with an average of 485 electrodes found to be speech responsive for each band. While some overlap was observed in the sites categorized as speech responsive across bands, the majority of speech responsive sites were speech responsive for only one band. This can be seen in Supplementary Figure 2. Similarly, significant sites for the tap, regular past tense, and regular plural comparisons were overwhelmingly band-specific (Supplementary Figure 3).

Supplementary Note 3

The null distribution for each of the neural response bands was generated from the comparison of random pairs of phones (i.e., A, B) as described in Results Section 'Acoustics, phonology, and morphology drive neural activity' and Methods Section 'Significant Electrodes'. However, gross featural and environmental properties of these randomly chosen pairs differ from those of the linguistically meaningful pairs in ways that could impact the suitability of the generated distribution as a null distribution for assessing the significance of the linguistically meaningful pairs. In this section, we confirm that distributions of randomly chosen pairs of sounds with featural and environmental properties similar to those of the linguistically meaningful pairs are not distinguishable from the overall null distribution. In doing so, we confirm that the distribution generated from random pairs of phones is a suitable null distribution.

The first property of the randomly chosen pairs that distinguishes them from the linguistically meaningful pairs is that the randomly chosen pairs are on average more featurally different from one another than the linguistically meaningful pairs. In other words, the large numbers of significant sites for the linguistically meaningful comparisons could be driven by the phonological similarity of the comparisons /t/ vs /d/ and /s/ vs /z/, each of which differ only in the feature [±voice]. If featural similarity drove the significance of the linguistically meaningful comparisons, we would expect the significance counts for random pairs (A,B) with fewer featural differences to be closer to the outer edge of the null distribution than the counts for random pairs with more featural differences. However, this is not the case. As shown in Supplementary Figure 4 the distribution of significant counts for random pairs with a single feature difference is not meaningfully distinct from the distribution of significant counts for random pairs with ten feature differences.

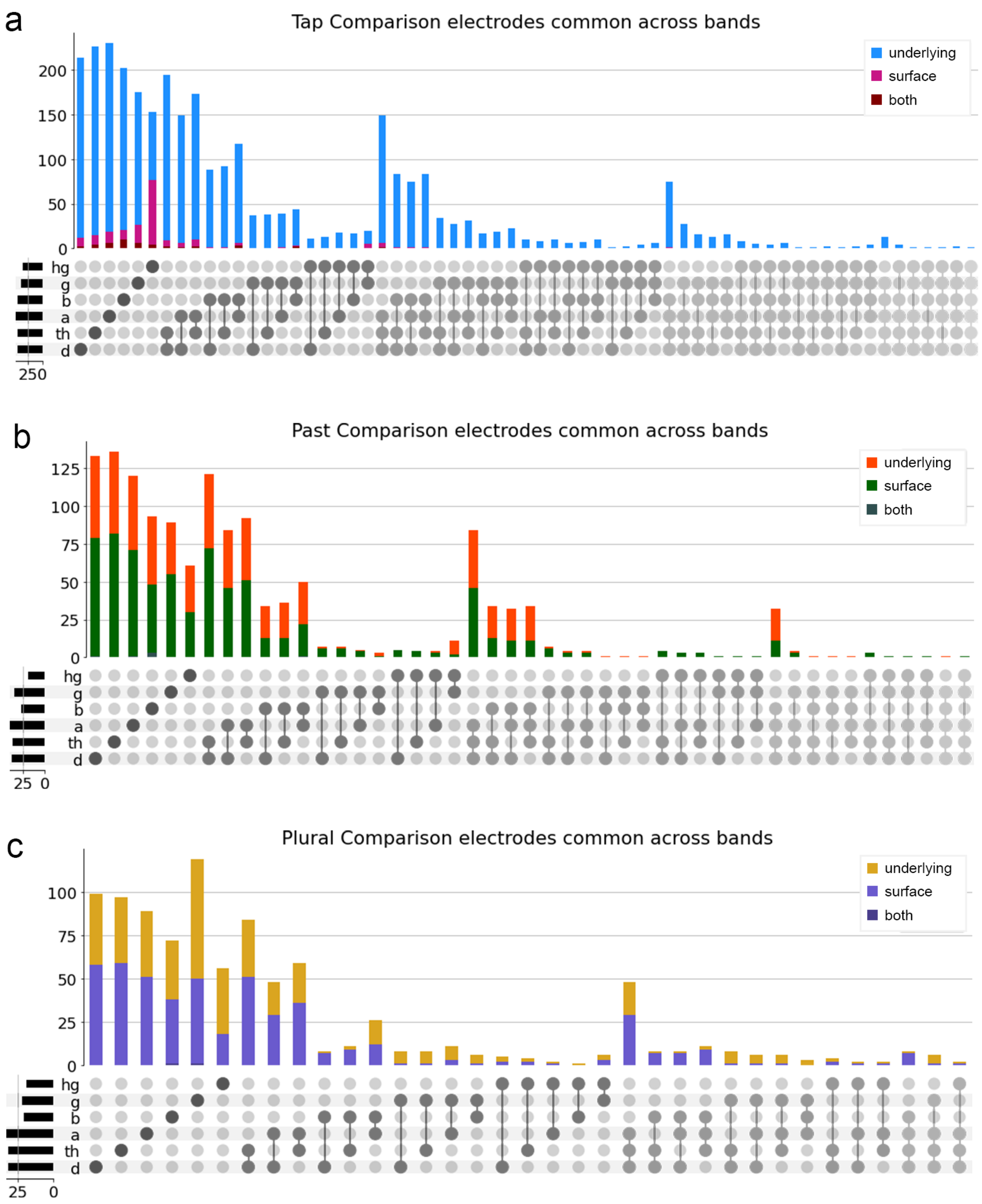

Supplementary Figure 3. **Degree of overlap across bands for channels identified as significant for each of the three (morpho)phonological comparisons.** For each subfigure, lower dot matrix plots indicate the combination of bands being considered, and upper bar plots show the number of speech responsive channels shared in common for that combination of bands. Combinations of bands not indicated in the dot matrix plots shared no significant sites in common. **a** Number of sites identified as acoustic (pink), phonemic (blue) or both (red) by the coronal tap alternation. **b** Number of sites identified as surface (green), morphological (orange), or both (dark green) by the regular past tense alternation. **c** Number of sites identified as surface (purple), morphological (gold), or both (dark purple) by the regular plural alternation.

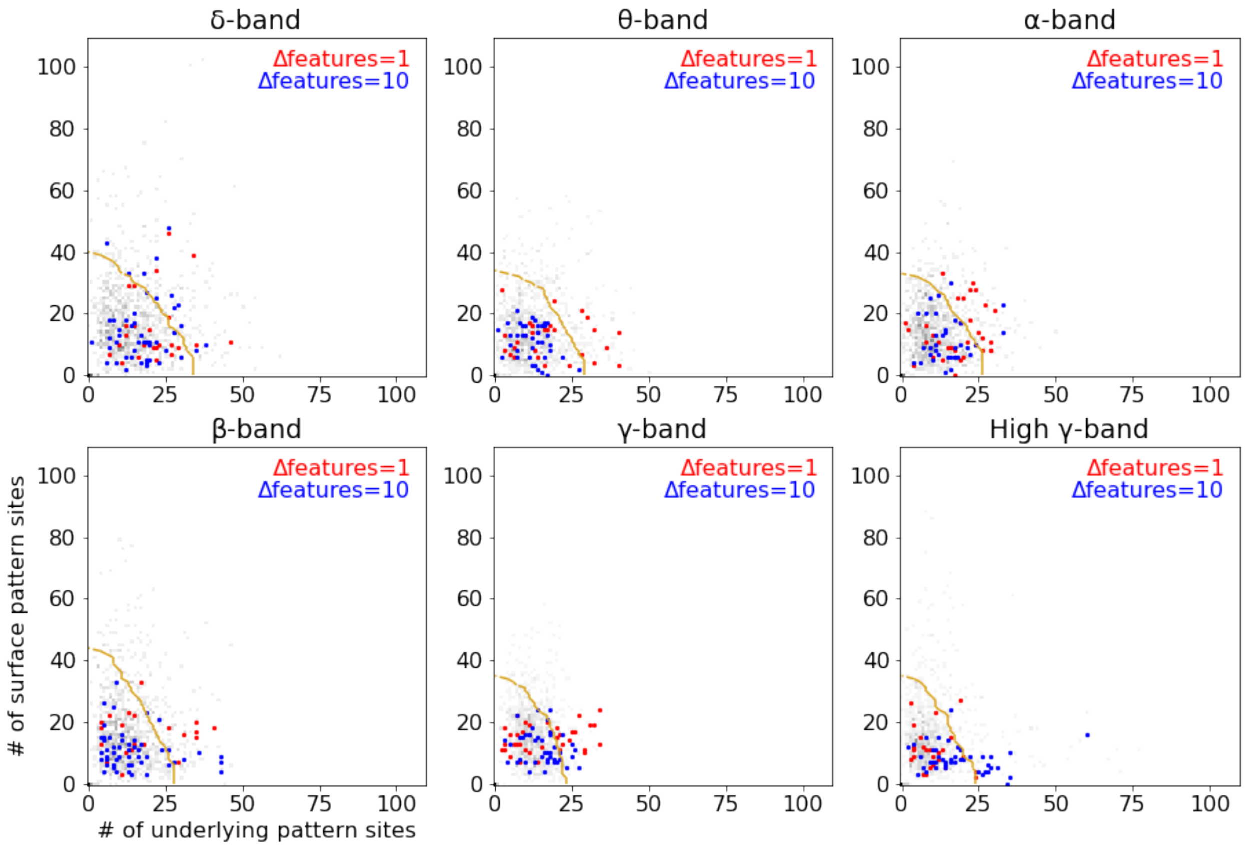

Supplementary Figure 4. **Distance in feature changes between phones does not structure the null distribution.** Number of significant sites observed for random pairs differing in one phonological feature[12] (red) and ten phonological features (blue) for each neural response band, relative to the remainder of the generated null distribution (gray). Vertical axes indicate the number of sites selective for surface identity observed for each comparison, while horizontal axes indicate the number of sites selective for underlying identity observed for each comparison. Dashed gold lines delimit the boundary containing 95% of the null distribution.

The second property of the randomly chosen pairs that distinguishes them from the linguistically meaningful pairs is the consistency of their phonological environments. That is, while all sounds considered in the plural and past tense comparisons were word-final sounds, pairs in the null distribution were not position-restricted. If the consistency in the phonological environment surrounding the phones participating in the tap, plural, and past tense alternations drove the number of significant sites observed for those comparisons, then we would expect comparisons of random phones in consistent phonological environments to accompany similarly large numbers of significant sites. To assess this, we performed the A, B_x, B_y analysis on 25 pseudo-random pairs of word-initial phones (i.e., all word-initial [z] vs. all word-initial [s]) and 25 pseudo-random pairs of word-final phones (i.e., all word-final [n] vs. all word-final [m]). Again, the distributions of randomly-chosen phones with consistent phonological environments falls well within the overall distribution of all randomly-chosen phones, as shown in Supplementary Figure 5.

Supplementary Note 4

In the main body of the manuscript, for a site to be considered an acoustic site for the coronal tap comparison, there must have existed at least one time window with a significant difference between surface [t] and tap /t/ tokens and between surface [t] and tap /d/ tokens but no significant difference

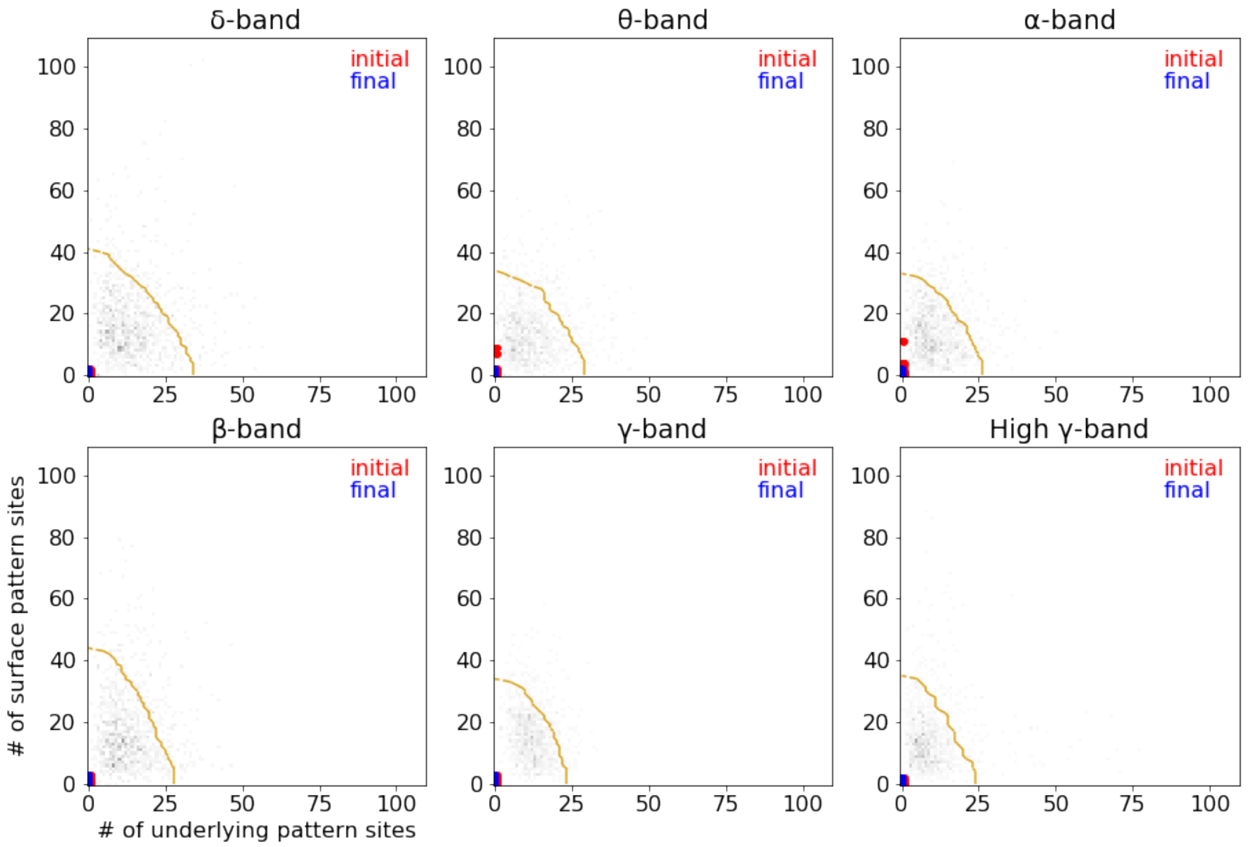

Supplementary Figure 5. **Consistency in phonological environment does not structure the null distribution.** Number of significant sites observed for 25 random pairs restricted to word-initial position (red) and word-final position (blue) for each neural response band, relative to the remainder of the generated null distribution (gray). Values have been jittered by <1 unit so that all unique values are visible. Vertical axes indicate the number of sites selective for surface identity observed for each comparison, while horizontal axes indicate the number of sites selective for underlying identity observed for each comparison. Dashed gold lines delimit the boundary containing 95% of the null distribution.

between tap /t/ and tap /d/ tokens. For a site to be considered a phonemic site, there must have existed at least one time window with a significant difference between tap /d/ and tap /t/ tokens and between tap /d/ and surface [t] tokens but no significant difference between surface [t] and tap /t/ tokens. We say that these comparisons have a "/t/ anchor" because they compare two kinds of /t/ with one kind of /d/.

A priori, the /t/ anchor comparison was chosen for analysis because surface realizations of /t/ (e.g., [t], [t^h]) are generally more acoustically distinct from taps than surface [d], and we wanted it to be particularly unlikely that underlying sites for the tap comparison (those grouping [t]/[t^h] with /t/-taps) could be explained away as another kind of acoustic similarity. However, the comparisons could also be done with a /d/ anchor. Then, for a site to be considered an acoustic site for the coronal tap comparison, there must have existed at least one time window with a significant difference between surface [d] and tap /d/ tokens and between surface [d] and tap /t/ tokens but no significant difference between tap /d/ and tap /t/ tokens. For a site to be considered a phonemic site, there must have existed at least one time window with a significant difference between tap /t/ and tap /d/ tokens and between tap /t/ and surface [d] tokens but no significant difference between surface [d] and tap /d/ tokens. The difference between /t/-anchor and /d/-anchor comparisons for the coronal tap alternation are shown in

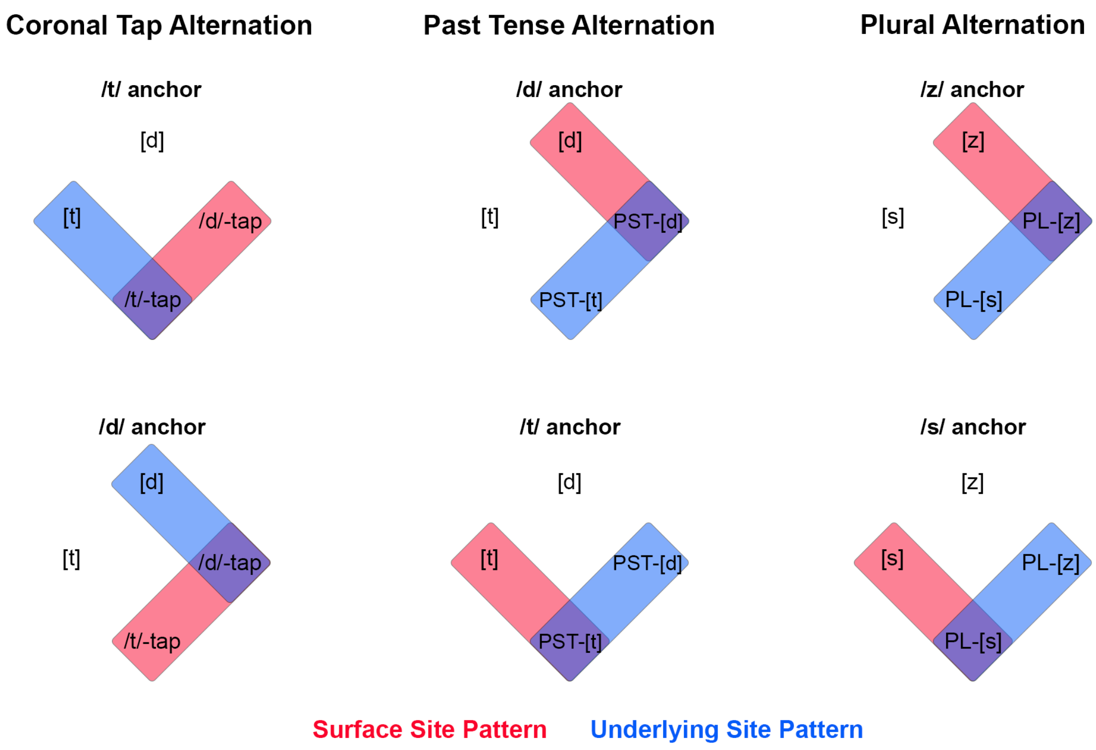

Supplementary Figure 6. **Schematic illustration of surface site and underlying site patterns for both anchors for each of the three (morpho)phonological comparisons.** Each column shows a different comparison: coronal tap (left), past tense (center), plural (right). The top row shows the anchors described in the main body of the manuscript, and the bottom row shows the alternate anchors. For surface sites, the sounds grouped together in red pattern together to the exclusion of the sound in blue. For underlying sites, the sounds grouped together in blue pattern together to the exclusion of the sound in red. Only three sounds are compared for each anchor.

the lefthand column of Supplementary Figure 6. The top row contains the anchors reported in the main body of the manuscript.

Similarly, the comparisons reported in the main body of the manuscript for the past tense and plural are /d/-anchor and /z/-anchor patterns, as illustrated in the top row of Supplementary Figure 6. These anchors were chosen *a priori* because they are typically considered to be the underlying forms of the regular past and plural morphemes, respectively. However, /t/ and /s/ anchors are also possible, as illustrated in the bottom row of Supplementary Figure 6.

Both anchors for each of the three comparison types result in meaningful comparisons for assessing surface and underlying similarity. For this reason, it is predicted that all six comparisons should result in significant numbers of surface and underlying sites, and indeed this is the case. The numbers of surface and underlying sites for each of the six comparisons are plotted relative to the generated null distribution in Supplementary Figure 7.

Supplementary Note 5

Results of this study do not support underspecification theories of phonology. If we assume, uncontroversially, that the featural difference between /t/ and /d/ and the difference between /s/ and /z/ is their value for the feature [$\pm$voice], then we would predict that surface sites for the past tense comparison, where tokens of /t/ are distinguished from /d/ regardless of their morphological content, would be the same as surface sites for the plural comparison, where tokens of /s/ and /z/ are distinguished. However, across all subjects and bands, only 2% of surface sites are shared between the past tense

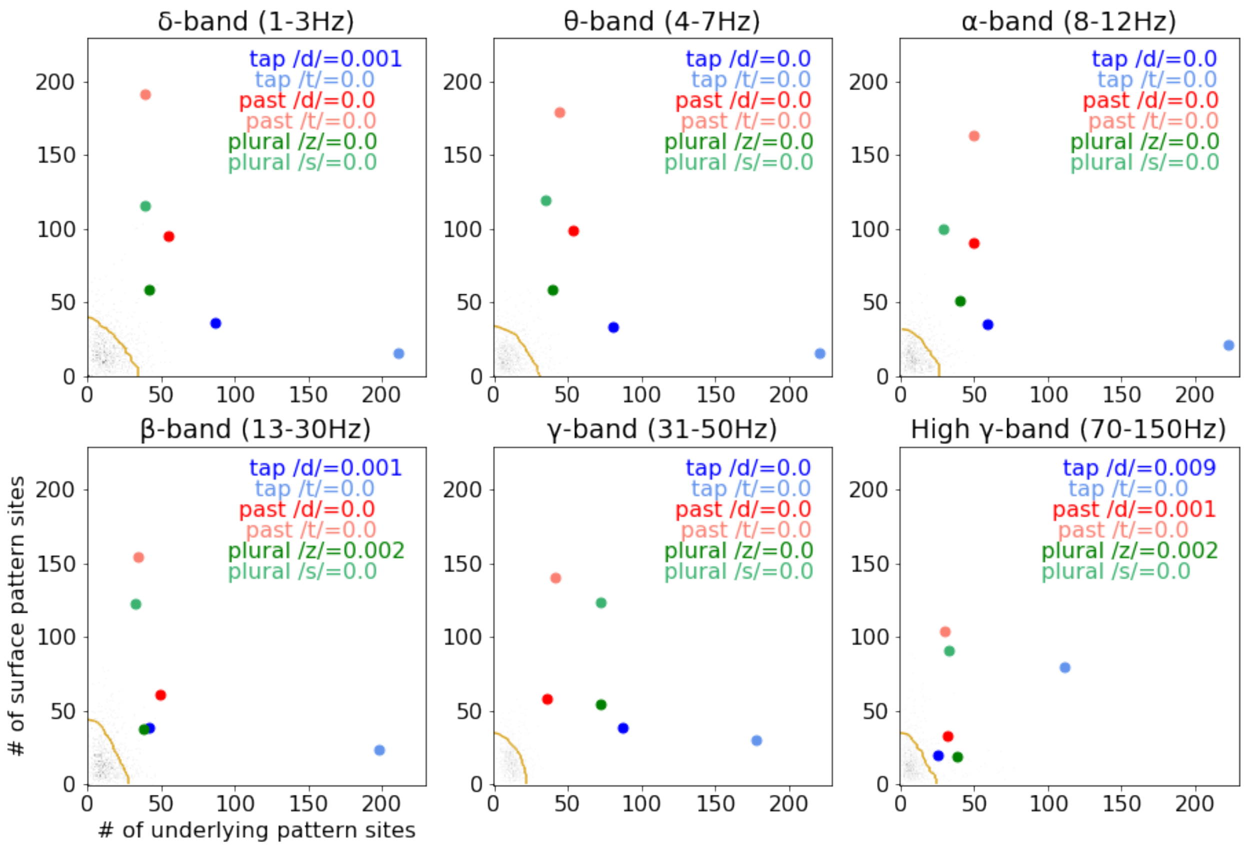

Supplementary Figure 7. **Surface similarity and underlying similarity patterns are not random.** Number of significant sites observed for both anchors of each (morpho)phonological comparison for each neural response band relative to the generated null distribution for that band. Each null distribution (greys) contains 1,000 comparisons. Vertical axes indicate the number of sites selective for surface identity observed for each comparison, while horizontal axes indicate the number of sites selective for underlying identity observed for each comparison. The proportion of the null distribution that contains at least as many surface and underlying sites as were observed for each comparison is indicated in the top right corner of each plot. Values for the tap comparison are blue; values for the past tense comparison are red; and values for the plural comparison are green. The anchor for each comparison is given within the slashes. Dashed gold lines delimit the boundary containing 95% of the null distribution.

and plural comparisons, as shown in Supplementary Figure 8. Moreover, when phonemic sites identified from the tap comparison, in which tokens of /d/ and /t/ are also hypothetically distinguished by the single feature [±voice], are considered alongside plural and past tense surface sites, the number of sites shared in common drops to two. Thus, it is highly unlikely that these sites index the presence or absence of the feature [±voice], since we would not expect the addition of phonemic sites identified by the tap comparison to further exclude candidate [±voice] sites that were identified by the past tense and plural surface comparisons. In this way, the findings of this study compel careful reconsideration of underspecification theories in phonology.

Supplementary Note 6

For the coronal tap alternation, the time windows with a significant acoustic or phonemic response varied across sites. For both the acoustic and phonemic response patterns, each time window was significant for at least one site in at least one band, with the exception of the 0–100ms window. In delta, theta, and

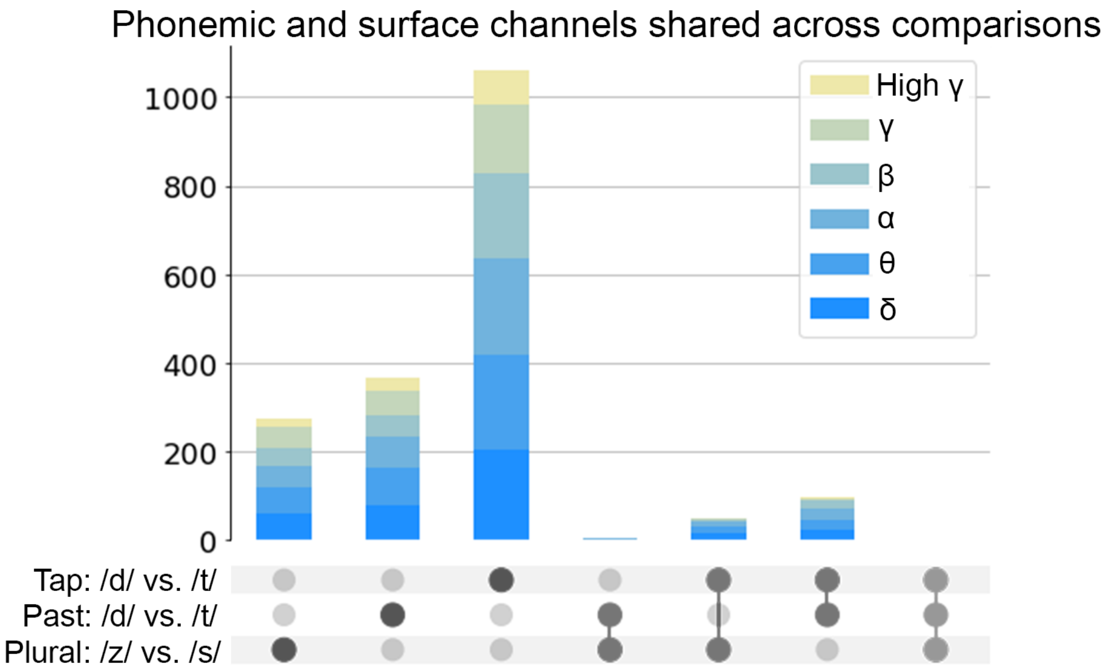

Supplementary Figure 8. **Phonemic and surface channels shared across comparisons, organized by response band.** The lower dot matrix plot indicates the combination of comparisons being considered, and the upper bar plot shows the number of significant channels shared in common for that combination of bands. Colors within each bar indicate the distribution of significant channels across response bands.

alpha bands, the greatest numbers of underlying sites were significant in the 200–300ms window, and in the beta band, the greatest number of underlying sites were significant in the 350–400ms window. Across all bands, the majority of surface sites were significant between 100–250ms. The distributions of surface and underlying sites are most closely coupled in the high gamma band. The distributions of significant time windows for these response patterns is shown in the top row of Supplementary Figure 9.

Similar to the coronal tap comparison, significant time windows for both the past tense and plural comparisons varied substantially across sites. For the plural comparison, in delta, theta, alpha, and beta bands, the greatest numbers of underlying sites were significant in the 150–200ms window. However, similar patterns are not apparent for the past tense comparison, and in general the basic temporal distribution of significant sites for the two morphological comparisons is less immediately interpretable than that of the coronal tap comparison. The full distributions of significant windows for past tense and plural comparisons are also shown in Supplementary Figure 9.

Supplementary Note 7

Locations of significant sites are spread bilaterally throughout fronto-temporal areas, as shown in Supplementary Figure 10. However, given the paucity of coverage over posterior cortices, the broad distribution of significant sites in traditional language areas is not a positive result; rather, without further corroboration from whole brain imaging studies, this distribution can be conservatively considered artifactual of commonalities among clinical presentations that require invasive neuromonitoring. Similarly, the larger number of significant sites in right hemisphere areas should not be taken as evidence for the lateralization of any effect, since more sites were recorded from right hemisphere than from the left. In general,

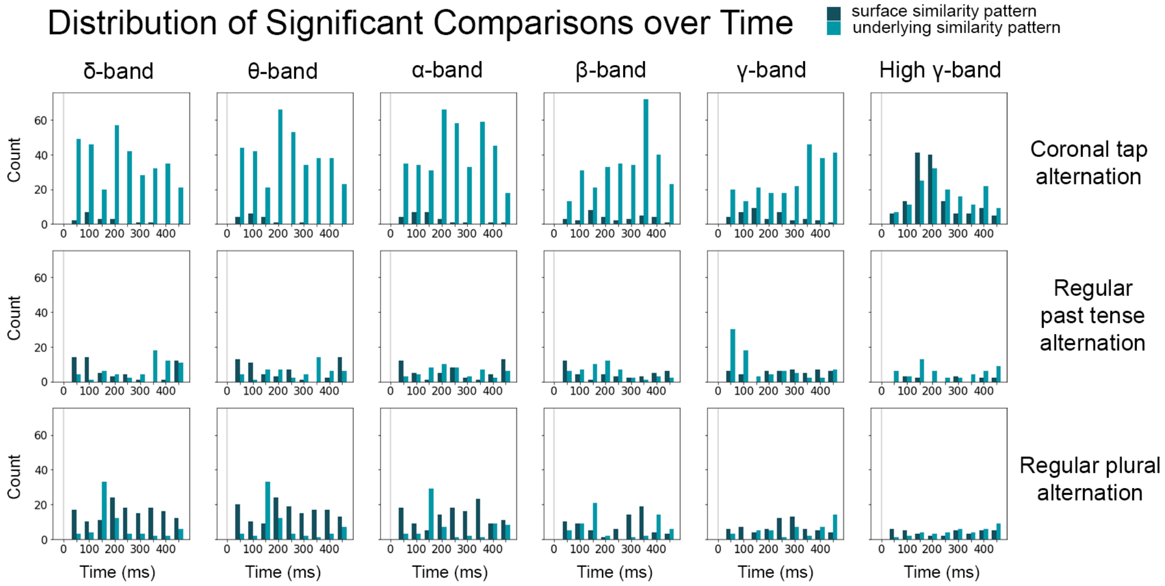

Supplementary Figure 9. **Distribution of significant channels over time.** For each neural response band (columns) and each linguistic comparison (rows), plots show the number of channels exhibiting surface and underlying responses for each time bin of the sliding window ANOVA. Bins correspond to 100ms windows beginning with the value indicated on the plot's horizontal axis. For each time bin, two bars are present: the navy bar shown on the right of each bin counts the number of sites selective for surface identity (i.e., 'acoustic' sites for the tap comparison and 'surface' sites for the plural and past tense comparisons), while the teal bar on the left counts the number of sites selective for underlying identity (i.e., 'phonemic' sites for the tap comparison and 'morphological' sites for the plural and past tense comparisons)

due to the clinically motivated biases inherent in the distribution of channel locations, this work does not make any claims about the relative distributions of channels based on any number of factors (e.g. response band, linguistic comparison, etc.) because such claims cannot be responsibly substantiated given the data and analyses used in this paper. For this reason, information about the localization of various electrodes is presented purely discriptively, to aid in cross-referencing with effects found in future work.

For both surface and underlying patterns for all three linguistic comparisons, the majority of significant electrodes are located in white matter. For the coronal tap alternation, acoustic and phonemic sites are diffusely distributed, and do not cluster separately from one another, as shown in Supplementary Figure 10a. Similarly, surface and morphological sites for the plural and past tense alternations do not cluster separately from one another and are broadly distributed across recorded sites, as shown in Supplementary Figure 10b,c. These general properties hold across bands, and over the full time course of the neural response. That is, it is not the case that clusters of sites identified as either surface or underlying emerge in particular bands or at particular times. While many aspects of language processing are characterized as distributed, for phonological phenomena, the degree of distribution observed here is uncharacteristic, and likely attributable to underdocumented properties of white matter in linguistic processing.

Supplementary Note 8

In addition to using a spectrographic feature set comprising 128-dimensional representations of the preceding 512ms of context, LME models were also fit using 128-dimensional representations of 1024ms

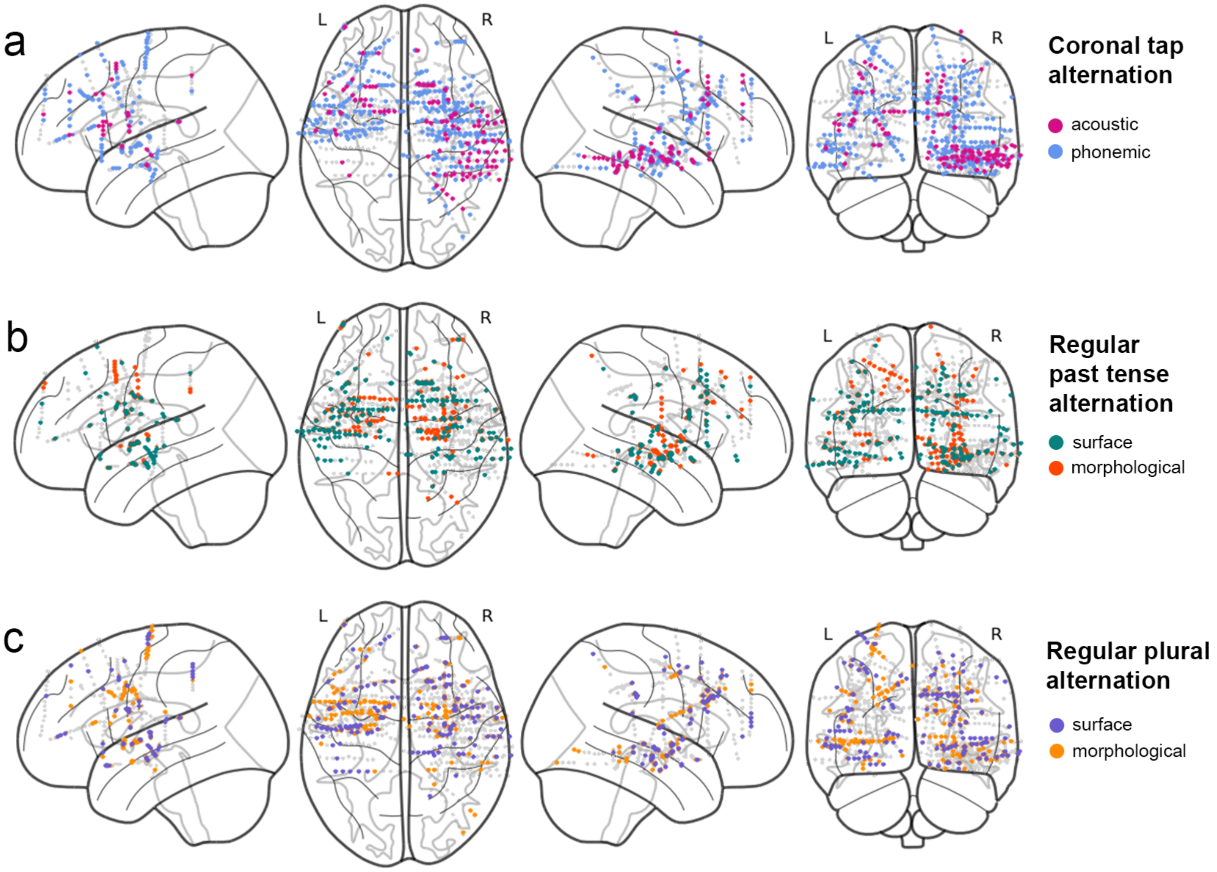

Supplementary Figure 10. **Distributions of significant sites for all neural response bands for nine participants.** **a** Locations of acoustic (pink) and phonemic (blue) sites identified by the coronal tap alternation. **b** Locations of surface (green) and morphological (orange) sites identified by the regular past tense alternation. **c** Locations of surface (purple) and morphological (gold) sites identified by the regular plural alternation. All subfigures were created using the Python package `nilearn` (DOI: 10.5281/zenodo.8397156).

spectrograms that had been compressed by a factor of two using a Generative Adversarial Interpolative Autoencoder (GAIA)[13] that had been trained on the original spectrograms.

For each participant, for each of the seven neural response types (=six classic frequency bands and broadband LFP), seven LME models were fit. Each model fit neural response with electrode channel and excerpt speaker as random effects and either only spectrographic features, only phonemic label features, or both spectrographic and phonemic label features as fixed effects. Supplementary Figure 11 illustrates how these three base models where constructed. Four additional models were also created, in which the phonemic or spectrographic features had either been shuffled within each excerpt or shuffled across the full recording session. Models were then compared within participant and neural response type using the Akaike Information Criterion (AIC),[14] and all best-fit models carried 100% of the cumulative model weight and had an AIC score >200 lower than other models.

Broadband LFP as well as power in delta, theta, alpha, and beta bands were best fit by linear mixed effects models that included both spectrographic features and phonemic labels (**s1p1**). For these bands, the **s1p1** model was the best fit for all participants. These results suggest that power in these bands is driven in part by phonemic category information that is not reducible to speech acoustics. For power

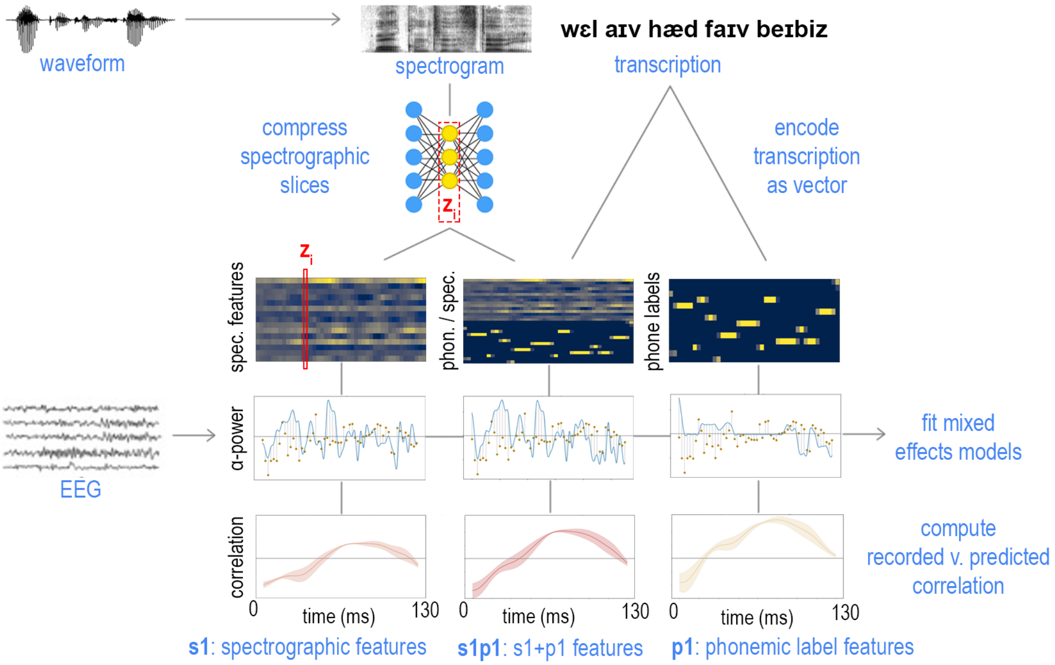

Supplementary Figure 11. **LME approach models neural activity as a combination of spectrographic and phonemic label features.** For each stimulus waveform, spectrograms are computed and time-aligned phonemic-level transcriptions are assigned (top row). Spectrograms are compressed into 128-dimensional vectors using a generative adversarial autoencoder network, and transcriptions are one-hot encoded. Three classes of model are created from these features: a purely spectrographic model (left column), a purely phonemic label model (right column), and a model containing both feature sets (middle column). For each band of neural activity, mixed effects models are fit. Model weights are used to reconstruct a predicted response for each band, and the correlation between the predicted and recorded neural response is calculated.

in gamma and high-gamma bands, however, the best fit model varied across individuals. For gamma power, eight participants' data were best fit by the model that included only spectrographic features (**s1**), and two participants' data were best fit by the **s1p1** model that included both spectrographic and phonemic label features (SD013, SD018). Similarly, for high-gamma power, eight participants' data were best fit by the **s1** model that included only spectrographic features, and the remaining participants data were best fit by the **s1p1** model that included both spectrographic and phonemic label feature sets (SD011, SD013). These results suggest that power in frequencies above 30Hz are primarily driven by speech acoustics rather than phonemic category information.

For each subject and response type, the model with the second lowest AIC score was >100 times as likely as the third ranked model. Models containing only phonemic label features were most likely to be the second ranked model for lower frequency bands. However, with increasing band frequency, the **s1** model becomes more likely to provide a better fit for the data until, for gamma and high-gamma bands, it is the best fit model overall. These results provide support for the generalization that phonemic labels better explain power at lower frequencies, while acoustic features excel at explaining power in higher frequencies.

Supplementary Note 9

AIC	Delta	AICw	Model Fixed Effects
-274494.0	0.00	1.00	`model + label + model:label + shuffle + band`
-274439.2	54.75	0.00	`label + shuffle + band`
-274437.8	56.35	0.00	`model + label + shuffle + band`
-274348.0	145.92	0.00	`shuffle + band`
-274346.4	147.52	0.00	`model + shuffle + band`

Supplementary Table 1. **Fixed effects and AIC scores for MNE models.** Models arranged in descending order of fit. Subject and channel were included as random effects for all models.

AIC	Delta	AICw	Model Fixed Effects
-1703033	0.00	1.00	`lang + model + lang:model + band`
-1702423	609.59	0.00	`lang + model + band`
-1637913	65120.24	0.00	`model + band`

Supplementary Table 2. **Fixed effects and AIC scores for mixed models that were fit on r^2 values for the correlation between the neural responses predicted by LME models and those recorded in actuality.** Models arranged in descending order of fit. Subject and channel were included as random effects for all models.

AIC	Delta	AICw	Model Fixed Effects							
			`band`	`mod`	`lab`	`mod:lab`	`lang`	`lang:mod`	`lang:lab`	`lang:mod:lab`
-313838.1	0.00	1.00	✓	✓	✓	✓	✓	✓	✓	✓
-313062.2	775.87	0.00	✓	✓	✓	✓	✓	✓	✓	
-313057.1	780.96	0.00	✓	✓	✓	✓	✓		✓	
-312431.7	1406.36	0.00	✓	✓	✓	✓	✓	✓		
-312426.7	1411.37	0.00	✓	✓	✓	✓	✓			
-312406.0	1432.05	0.00	✓	✓	✓	✓				

Supplementary Table 3. **Fixed effects and AIC scores for mixed models that were fit on r^2 values for the correlation between the neural responses predicted by MNE models and those recorded in actuality.** Models arranged in descending order of fit. Subject and channel were included as random effects for all models. Model fixed effects are frequency band (`band`; delta, theta, alpha, beta, gamma, or high gamma), model type (`mod`; linear or quadratic), label status (`lab`; labeled or unlabeled), language (`lang`; English or Catalan), and their interactions.

References

[1] Zue, V. W. & Laferriere, M. Acoustic study of medial /t, d/ in American English. *J. Acoust. Soc. Am.* **66**, 1039–1050 (1979).

[2] Braver, A. Incomplete neutralization in American English flapping: A production study. *Proc. Mtgs. Acoust.* **19** (2013).

[3] Derrick, D. & Schultz, B. Acoustic correlates of flaps in North American English. In *Proceedings of Meetings on Acoustics ICA2013*, vol. 19 (Acoustical Society of America, 2013).

[4] Hillenbrand, J., Ingrisano, D. R., Smith, B. L. & Flege, J. E. Perception of the voiced–voiceless contrast in syllable-final stops. *J. Acoust. Soc. Am.* **76**, 18–26 (1984).

[5] Hogan, J. T. & Rozsypal, A. J. Evaluation of vowel duration as a cue for the voicing distinction in the following word-final consonant. *J. Acoust. Soc. Am.* **67**, 1764–1771 (1980).

[6] Revoile, S., Pickett, J. M., Holden, L. D. & Talkin, D. Acoustic cues to final stop voicing for impaired-and normal hearing listeners. *J. Acoust. Soc. Am.* **72**, 1145–1154 (1982).

[7] Charles-Luce, J. Cognitive factors involved in preserving a phonemic contrast. *Lang. Speech* **40 (Pt 3)**, 229–248 (1997).

[8] Fox, R. A. & Terbeek, D. Dental flaps, vowel duration and rule ordering in american english. *J. Phon.* **5**, 27–34 (1977).

[9] Sharf, D. J. Duration of post-stress intervocalic stops and preceding vowels. *Lang. Speech* **5**, 26–30 (1962).

[10] van der Walt, S. *et al.* Scikit-image: Image processing in Python. *PeerJ* **2**, e453 (2014).

[11] Herd, W., Jongman, A. & Sereno, J. An acoustic and perceptual analysis of /t/ and /d/ flaps in American English. *J. Phon.* **38**, 504–516 (2010).

[12] Riggle, J. Phonological feature chart (2011). URL `https://artoflanguageinvention.com/papers/features.pdf`.

[13] Sainburg, T., Thielk, M., Theilman, B., Migliori, B. & Gentner, T. Generative adversarial interpolative autoencoding: adversarial training on latent space interpolations encourage convex latent distributions. *arXiv preprint arXiv:1807.06650* (2018).

[14] Akaike, H. Information theory and an extension of the maximum likelihood principle. In *Selected papers of Hirotugu Akaike*, 199–213 (Springer, 1998).
