# Supplementary material for: Acoustic and language-specific sources for phonemic abstraction from speech
Source: Nat Commun. 2024 Jan 23;15:677. doi: 10.1038/s41467-024-44844-9 (PMC10805762; doi:10.1038/s41467-024-44844-9)
Supplement: Supplementary file 3 — Reporting Summary [file 41467_2024_44844_MOESM3_ESM.pdf]

nature portfolio

Corresponding author(s): Anna Mai

Last updated by author(s): 3 November 2023

Reporting Summary

Nature Portfolio wishes to improve the reproducibility of the work that we publish. This form provides structure for consistency and transparency in reporting. For further information on Nature Portfolio policies, see our Editorial Policies and the Editorial Policy Checklist.

Statistics

For all statistical analyses, confirm that the following items are present in the figure legend, table legend, main text, or Methods section.

n/a	Confirmed	
☐	☒	The exact sample size (*n*) for each experimental group/condition, given as a discrete number and unit of measurement
☒	☐	A statement on whether measurements were taken from distinct samples or whether the same sample was measured repeatedly
☐	☒	The statistical test(s) used AND whether they are one- or two-sided<br>*Only common tests should be described solely by name; describe more complex techniques in the Methods section.*
☐	☒	A description of all covariates tested
☐	☒	A description of any assumptions or corrections, such as tests of normality and adjustment for multiple comparisons
☐	☒	A full description of the statistical parameters including central tendency (e.g. means) or other basic estimates (e.g. regression coefficient) AND variation (e.g. standard deviation) or associated estimates of uncertainty (e.g. confidence intervals)
☐	☒	For null hypothesis testing, the test statistic (e.g. *F*, *t*, *r*) with confidence intervals, effect sizes, degrees of freedom and *P* value noted<br>*Give P values as exact values whenever suitable.*
☒	☐	For Bayesian analysis, information on the choice of priors and Markov chain Monte Carlo settings
☐	☒	For hierarchical and complex designs, identification of the appropriate level for tests and full reporting of outcomes
☐	☒	Estimates of effect sizes (e.g. Cohen's *d*, Pearson's *r*), indicating how they were calculated

Our web collection on statistics for biologists contains articles on many of the points above.

Software and code

Policy information about availability of computer code

Data collection	Stimuli were presented using PsychoPy for Python 2.7. EEG data were collected using Natus NeuroWorks Software
Data analysis	Open source Python (3.6.5) and R (3.6.3) libraries were used to analyze the data in this study. Standard functions in MNE Python (0.20.8) were used for data preprocessing. LME models were constructed and fit using the R library lme4 (1.1-21). MNE models were constructed and fit using the Python package pyMNE (https://github.com/MarvinT/pyMNE).

For manuscripts utilizing custom algorithms or software that are central to the research but not yet described in published literature, software must be made available to editors and reviewers. We strongly encourage code deposition in a community repository (e.g. GitHub). See the Nature Portfolio guidelines for submitting code & software for further information.

Data

Policy information about availability of data

All manuscripts must include a data availability statement. This statement should provide the following information, where applicable:

- Accession codes, unique identifiers, or web links for publicly available datasets
- A description of any restrictions on data availability
- For clinical datasets or third party data, please ensure that the statement adheres to our policy

The datasets generated and analyzed during the current study are available in the OpenNeuro repository, registered with this DOI: doi:10.181112/openneuro.ds004703.v1.0.0.

Research involving human participants, their data, or biological material

Policy information about studies with human participants or human data. See also policy information about sex, gender (identity/presentation), and sexual orientation and race, ethnicity and racism.

Reporting on sex and gender	The gender of each participant was assigned visually and impressionistically by the corresponding author during their initial encounter with the participant. Sex/gender information for each participant was also communicated by the participant's clinical team prior to their surgery. These assignment methods were consistent for all patients for this study. Four study participants were women, and six were men. Sex/gender did not play a determining role in whether patients were selected for inclusion in the study, nor were any sex- or gender-based analyses performed as part of this study, as n were too small.
Reporting on race, ethnicity, or other socially relevant groupings	No race, ethnicity, or other socially relevant group information was collected.
Population characteristics	Participants were 21-55 years old (mean=32), and all were diagnosed with epilepsy or related conditions requiring intracranial EEG monitoring. Participants reported normal hearing and performed within acceptable range on a battery of neuropsychological language tasks prior to surgery.
Recruitment	Participants were patients undergoing intracranial EEG monitoring for epilepsy. They were recruited for research participation through their neurologist (Dr. Jerry Shih) and gave written informed consent prior to surgery. All participants performed within acceptable range on a battery of neuropsychological language tests prior to surgery, suggesting that the presence of clinical conditions (such as epilepsy) did not have undue effects on their language processing. No known self-selection biases exist.
Ethics oversight	UC San Diego Institutional Review Board

Note that full information on the approval of the study protocol must also be provided in the manuscript.

Field-specific reporting

Please select the one below that is the best fit for your research. If you are not sure, read the appropriate sections before making your selection.

☒ Life sciences ☐ Behavioural & social sciences ☐ Ecological, evolutionary & environmental sciences

For a reference copy of the document with all sections, see nature.com/documents/nr-reporting-summary-flat.pdf

Life sciences study design

All studies must disclose on these points even when the disclosure is negative.

Sample size	Number of participants was arbitrarily pre-established to be ten. Results are qualitatively consistent across all participants suggesting that this sample is not anomalous for this population (patients undergoing intracranial neuromonitoring). Stimuli were designed so that each participant would listen to at least 100 tokens of each speech sound of interest. The number 100 was determined semi-arbitrarily, through comparison with sample sizes used in other studies with similar designs. In particular, Boudewyn et al. (2017, Psychophysiology) show that depending on the ERP component(s) anticipated, anywhere from 6-90 repetitions per condition can be sufficient to reliably detect effects. Since we did not have strong a priori hypotheses for what components these specific speech sounds would elicit, the sample sizes for tokens of speech sounds were thus chosen to maximize the chance of observing relevant effects.
Data exclusions	Data containing epileptic activity were excluded from analysis because epileptic activity was assumed not to reflect normative language activity. This exclusion criterion was pre-established.
Replication	Reported within-subjects effects were qualitatively robust across participants with the exception of the effects reported in Figure 5A and discussed in Section 4. No other reproducibility measures have been taken. No experiments have been replicated at this time.
Randomization	Participants were not assigned to different groups. All comparisons were within-subjects.
Blinding	Investigators were not blind to the within-subjects conditions. Stimuli conditions were speech sound categories that were presented to participants as part of natural speech. Investigators were not blinded during data collection or analysis because when listening to natural speech, speech comprehension is more salient than individual speech sound segregation (i.e., investigators cannot keep track of individual speech sound categories during the course of natural speech).

Reporting for specific materials, systems and methods

We require information from authors about some types of materials, experimental systems and methods used in many studies. Here, indicate whether each material, system or method listed is relevant to your study. If you are not sure if a list item applies to your research, read the appropriate section before selecting a response.

Materials & experimental systems

n/a	Involved in the study
☒	☐ Antibodies
☒	☐ Eukaryotic cell lines
☒	☐ Palaeontology and archaeology
☒	☐ Animals and other organisms
☒	☐ Clinical data
☒	☐ Dual use research of concern
☒	☐ Plants

Methods

n/a	Involved in the study
☒	☐ ChIP-seq
☒	☐ Flow cytometry
☐	☒ MRI-based neuroimaging

Plants

Seed stocks: n/a

Novel plant genotypes: n/a

Authentication: n/a

Magnetic resonance imaging

Experimental design

Design type: structural anatomical scans

Design specifications: no experimental design; whole brain scans were collected for electrode localization only.

Behavioral performance measures: no behavioral performance measures were gathered; anatomical scans were collected for electrode location.

Acquisition

Imaging type(s): structural

Field strength: 3T

Sequence & imaging parameters: All scans were 3D T1-weighted ~1mm voxel isotropic, collected clinically. Scan protocol depended on the location where the scans were collected. GE scans were collected with FSPGR protocol, and Siemens scans were collected with MPRAGE protocol.

Area of acquisition: whole brain scan

Diffusion MRI: ☐ Used ☒ Not used

Preprocessing

Preprocessing software: Processing was carried out in FreeSurfer (v6), using the standard recon-all pipeline described in Fischel (2012). [Fischel, B. (2012) FreeSurfer. Neuroimage. 62:774-781.]

Normalization: All normalization procedures included in the standard recon-all pipeline were performed.

Normalization template: MNI305 normalization template was used, per standard execution of -talairach as part of standard recon-all pipeline.

Noise and artifact removal: All noise and artifact removal procedures included in the standard recon-all pipeline were performed.

Volume censoring: Volume censoring is not performed as part of the standard recon-all pipeline, and was not performed on these data.

Statistical modeling & inference

Model type and settings: No statistical modeling or inference was performed on MRI data for this study.

Effect(s) tested | No statistical modeling or inference was performed on MRI data for this study.

Specify type of analysis: ☐ Whole brain ☐ ROI-based ☐ Both

Statistic type for inference (See Eklund et al. 2016) | No statistical modeling or inference was performed on MRI data for this study.

Correction | No statistical modeling or inference was performed on MRI data for this study.

Models & analysis

n/a	Involved in the study
☒	☐ Functional and/or effective connectivity
☒	☐ Graph analysis
☒	☐ Multivariate modeling or predictive analysis
